# Communication with cancer patients: the perspective of caregivers versus non-caregivers in Iran

**DOI:** 10.3389/fpsyg.2023.1239410

**Published:** 2023-11-02

**Authors:** Azam Naghavi, Samaneh Salimi, Winfried Rief, Pia von Blanckenburg

**Affiliations:** ^1^Department of Counseling, Faculty of Education and Psychology, University of Isfahan, Isfahan, Iran; ^2^Department of Counseling, Faculty of Psychology and Education, University of Tehran, Tehran, Iran; ^3^Department of Clinical Psychology and Psychotherapy, Philipps University of Marburg, Marburg, Germany

**Keywords:** communication, cancer patients, breaking bad news, culture, caregiver

## Abstract

**Objective:**

This study investigated and compared the attitudes of healthy Iranian individuals (*n* = 302) in forms of two groups of caregivers and non-caregivers of cancer patients about the communication with cancer patients, and their personal wish to know the diagnosis if they ever had cancer. In addition, this study aimed to identify how many participants in the caregivers’ group had spoken with their family member affected by cancer about their illness.

**Methods:**

Caregivers (50.7%) and non-caregivers (49.3%) responded to two questionnaires regarding their general attitudes about communicating with cancer patients, and their willingness to know about their illness if they had cancer.

**Results:**

The majority of participants (92.8%), especially in the caregiver group, agreed with the right of patients to know the diagnosis and prognosis, and also wished to know if they ever had cancer. However, around 64% of caregivers never talked about cancer with the affected patients.

**Conclusion:**

Participants generally believed that patients have the right to know the diagnosis and prognosis, and they also wished to know if they ever had cancer. However, in reality many cancer patients are not included in communication sessions in Iran. Health professionals should focus on how to create a balance between medical bioethics with cultural influences on communication with patients.

## 1. Introduction

Communication with a patient about cancer is a challenging task for both healthcare providers and families. In some cases, it is the family that decides when, how, and how thoroughly to disclose the diagnosis and prognosis of cancer to the patient, and medical specialists may be requested to withhold bad news (Hume and Malpas, 2016). The principles of Beauchamp and Childress (2001) have far-reaching influence in medical ethics. In particular, the principle of “autonomy” has become the basis of legal regulations, and requests about “informed consent” (e.g., informing about potential side effects before starting medical interventions) and “shared decision making” with all its legal implications are rooted in this principle. While from an ethical perspective it can be discussed whether patients also have a right to “not knowing,” legal regulations in many countries have a clear preference for the interpretation that only fully informed patients can make autonomous decisions about how to proceed.

Several protocols have been designed to guide healthcare professionals to respect biomedical ethics and to communicate with patients about their diagnosis and treatment. For example, the SPIKES protocol, which is taught in many universities around the world, recommends that professionals follow six steps before breaking bad news. The steps include setting up the interview, assessing the patient’s perception, obtaining the patient’s invitation to hear the bad news, giving knowledge and information, addressing the patient’s emotions, strategies about the treatment, and summary (Baile et al., 2000). The SPIKES protocol is implemented moderately well (Seifart et al., 2014; von Blanckenburg et al., 2023; Wege et al., 2023). However, according to some studies, the protocol is may not culturally sensitive. In a survey of over 1,300 patients in Canada, Mirza et al. (2019) found that several patients’ needs were not included in the SPIKES protocol. In Middle Eastern (Farhat et al., 2015) and Asian (Shin et al., 2016; Hahne et al., 2020) countries, there is considerable resistance against direct communication with patients.

Before discussing the process of breaking bad news in the Iranian healthcare system, a small background about Iran worth mentioning. With approximately 80 million population, Iran is located in the Middle East and North Africa (MENA) region. The Iranian healthcare system consists of two main sectors: public and private. The Ministry of Health and Medical Education (MOHME) is the central authority that makes most of the decisions about the health system’s goals, policies and resources in Iran (Ministry of Health and Medical Education, 2023). Both public and private sector provide primary, secondary and tertiary healthcare. Even though the high-level Iranian documents (such as the third to sixth five-year development plans, Mega Health Policies) repeatedly stressed the importance of providing health insurance for all Iranians, lowering out of pocket health spending, and ensuring fair access to health care services, there are still challenges in health insurance coverage in Iran, and many patients have to pay the gap between public and private medical tariffs (Doshmangir et al., 2021). This can be specially challenging for high-cost treatments as occurs in cancer treatment.

Medical education is entirely supervised by MOHME in public and private universities. Breaking the bad news and specifically SPIKES protocol is part of the medical education in Iran, however, according to Labaf et al. (2014), there is no national data on the delivery of bad news by Iranian doctors and their mastery of the necessary communication skills and the adequacy of training provided in universities. Although over 80% of Iranian healthcare specialists and patients had positive attitudes toward telling the truth to a patient (Zamani et al., 2011; Nasrollahi et al., 2022), a study shows that only 35% of patients were completely informed about their disease, and only 7% of patients were aware of the prognosis (Larizadeh and Malekpour-Afshar, 2007), especially when the patient is young or old. Medical students in Iran learn how to break bad news, however in practice, it is often the families that handle the news and the information (Larizadeh and Malekpour-Afshar, 2007; Scheidt et al., 2017). In a recent representative study from Pakistan, the majority of patients expressed a preference for their family members to receive the bad news initially (Shah et al., 2023). When the family is involved in the diagnosis stage, such as receiving test results, meeting with various specialists to reach a diagnosis, the possibility that the patient is left out of the decision-making process is very high (Scheidt et al., 2017). In such a situation, it is very common that the family and surrounding people request the doctor not to tell the patient the definitive diagnosis.

While several studies on delivering bad news have been conducted worldwide, it is crucial to investigate people’s attitudes toward disclosing such news to a patient. As mentioned earlier, the perspectives of healthcare staff on a patient’s right to know may not align with the family’s preferred approach to conveying bad news. The caregivers have an important role and often wish not to harm the patients (Scheidt et al., 2017). Nevertheless, it could be, that attitudes about the communication process may change due to the experiences in the care and the communication compared to persons without any contact to the illness of cancer. Thus, it is essential to ascertain the personal preferences of caregivers regarding their own potential diagnoses. Do they wish to be informed of the diagnosis, or would they prefer not to know? Does the experience of caring for someone with cancer change one’s own attitudes? Are there any disparities between their attitudes and the reality? Considering the substantial number of Iranian migrants dispersed globally, along with the healthcare system in Iran, the findings of this study may hold global significance in ensuring culturally sensitive care. In this study, we aim to address the following questions:

1. What are the attitudes of healthy individuals (both caregivers and non-caregivers) toward communicating with cancer patients?
2. Who should inform about the illness if participants are diagnosed with cancer?
3. Is there a correlation between personal preferences for knowledge and general attitudes toward communicating with cancer patients? Are there any moderators to consider?
4. Among the caregivers, how many have had discussions about the disease with their family members affected by cancer?

Our hypothesis is, that there will be a significant difference in the attitudes toward communication with cancer patients between healthy individuals who are caregivers and those who are not due to the experience of caring for someone with cancer. We suppose, that there will be a correlation between participants’ personal treatment preferences and their general attitude toward communicating with cancer patients. The proportion of caregivers who have discussed the disease with their affected family member will be less than 50%.

## 2. Methods

### 2.1. Sample

The questionnaire was filled out by two groups of participants: caregivers and non-caregivers of cancer patients. Non-caregivers were recruited online through different social media, and caregivers were recruited online, through different social media (such as what’s up, Telegram and Instagram), and also from an NGO supporting people with cancer and their families. For online group, we formed an online questionnaire through google form and sent the link around. In the first page we wrote the statement of purpose and informed consent statement. Recruitment lasted for six months. Participants from the NGO were contacted by phone and if they agreed to participate, they were given an option between the online questionnaire or an interview via phone call. The majority of participants completed the online version. The data set was anonymous and is stored in a locked computer in the university. The study was approved by the University of Isfahan Ethics Committee (J/2509/99).

### 2.2. Instrument

Attitudes toward communication with cancer patients were assessed with an 18-item questionnaire that was piloted among 20 Iranians caregivers and non-caregivers. The questionnaire had two parts; the first part addressed attitudes of the participants about communicating with a cancer patient. In this part, six questions were selected and translated/ back translated from the German Marburg Breaking Bad News (MABBAN) scale (von Blanckenburg et al., 2020) which is a questionnaire that assesses patient preferences for breaking bad news communication based on the SPIKES protocol (Seifart et al., 2014). To ensure a broader approach (communication with the family/spouse as well) and not just communication with the doctor, the beginning of the items was changed from “The doctor should… e.g. give the patient the opportunity to ask questions” to “It is better to… e.g. give the patient the opportunity to ask questions”. Four items were developed by researchers and clinicians (e.g., “It is better that the patient knows that he/she has cancer”, “I want to know that I have cancer”) (see Table 1). All items were rated as agree or disagree. In the present study, the Cronbach’s alpha coefficient was estimated as 0.72. Guttman split half coefficient estimated as 0.79. The second part of the questionnaire started with the sentence “If I had cancer, I would want to know that I have cancer”, followed by the same items as the first part (“e.g. I want the following people to give me enough possibilities to ask questions”) with the preferred source of information (doctor, parents, children, spouse). Moreover, we asked the caregivers “Does anybody talk with the patient about his/her cancer?” and “Who was the person to break the bad news?” Guttman split half coefficient estimated as 0.78 for the second part. Content validity ratio (CVR) was assessed as well. We sent the questionnaire to eight health psychologist and received their responds about the necessity of the items. The CVR was calculated as 0.94 that was acceptable.

**Table 1:** Comparison of the general attitudes toward cancer communication among caregivers and non-caregivers.

		Caregivers (*N* = 153)	Non-caregivers (N = 149)	Chi^2^	
General attitudes about breaking bad news in cancer patients		Agree *n* (%)	Agree *n* (%)	Chi^2^	*p*-value
1	It is better that the patient knows that he/she has cancer.	127 (83)	133 (89.3)	2.57	0.12
2	It is better to talk to cancer patients about their illness.	128 (83.7)	133 (89.3)	2.02	0.15
3	It is better to inform the patient about the disease during the first conversation.	73 (47.7)	62 (41.6)	1.14	0.29
4	It is better to explain the details of the disease comprehensibly and in detail.*	117 (76.5)	119 (79.9)	1.14	0.29
5	It is better to give the patient enough possibilities to ask questions.*	150 (98.0)	147 (98.7)	0.18	0.67
6	It is better to inform the patient about possible therapies.*	149 (97.4)	147 (98.7)	0.63	0.43
7	It is better to inform the patient about alternative treatment methods (traditional therapy, palliative therapy).*	131 (85.6)	142 (95.3)	8.15	0.004
8	It is better to inform the patient about effects of the tumor on life circumstances.*	116 (75.8)	115 (77.2)	0.08	0.78
9	It is better to characterize the expected course of disease in all clarity.*	111 (72.5)	112 (75.2)	0.27	0.61
10	I want to know if I have cancer.	142 (92.8)	98 (65.8)	33.83	<0.001

Adapted from the MABBAN scale.

### 2.3. Statistical analyses

SPSS23 statistical software was used to analyze the data. Descriptive analyses were conducted to describe the sample’s demographic characteristics. Identifying the differences between two groups was tested using chi-squared tests. Logistic regressions with a moderated model (Model 1) were tested using the PROCESS macro to investigate the moderating effects of age, gender, education, and history in relation to the first and tenth questions of the questionnaire (Hayes, 2013).

## 3. Results

### 3.1. Demographic characteristics

Based on the Cochran’s (1977) formula and initial pilot scores (SD = 0.3), the general sample size was estimated to be at least 139 people. The questionnaire was completed by 302 participants; of these, 153 (50.7%) were caring for a cancer patient in their family, and 149 (49.3%) had no history of cancer in the family. Of this group, 250 individuals were female (82.8%) and 52 were male (17.2%). The age ranged between 30 and 65 years (M = 43.9, SD = 6.2). The majority of participants had a Bachelor’s degree (39/4%), followed by a Master’s degree (25/8%), high school diploma (17/9%), PhD (12/3%), and high school or less (4/8%).

### 3.2. Attitudes toward breaking Bad news

The frequency and percentage of participants’ answers to items 1 to 10 are presented in Table 1. In addition, the results of the chi-squared test are provided in Table 1 in order to determine significant differences in the frequency of answers by caregivers and non-caregivers. Most participants agreed with the general attitude that it is important to communicate with persons affected by cancer. The item “It is better to inform the patient about the disease during the first conversation” was agreed upon by less than half of the asked persons. Moreover, significantly fewer caregivers would inform the patient about alternative treatment methods. Interestingly, almost all caregivers (92.8%) would like to know if they were affected by cancer, but only two thirds of non-caregivers agreed with this statement (65%).

### 3.3. Who should inform about the disease

The results of the chi-squared test to examine the frequency of answers to questions 11 to 18 in the two groups are presented in Table 2. In response to all the questions of the questionnaire related to the person who informs the cancer patient of different aspects of the disease, between caregivers and non-caregivers, most people would prefer the doctor to inform them. In comparison between the two groups, only in response to question 14 (I want the following people to give me the opportunity to ask questions) was there a difference between groups (*p* < 0.05). More participants in the non-caregivers group were more likely than caregivers to have the opportunity to ask questions to their spouse.

**Table 2:** Who should inform about the illness (breaking bad news).

		Informant	Caregivers (*N* = 153)	Non-caregivers (*N* = 149)	Chi^2^	*p*-value
			*n* (%)	*n* (%)		
11	If I had cancer I want the following people to talk to me about my illness.	Doctor	127 (83)	119 (79.9)	2.10	0.55
		Parents	5 (3.3)	4 (2.7)		
		Children	1 (0.7)	0 (0)		
		Spouse	20 (13.1)	26 (17.4)		
12	I want the following people to inform me about the disease during the first conversation.	Doctor	117 (76.5)	116 (77.9)	1.28	0.73
		Parents	7 (4.6)	5 (3.4)		
		Children	1 (0.7)	0 (0)		
		Spouse	28 (18.3)	28 (18.8)		
13	I want the following people to explain me the details of the disease comprehensibly and in detail.	Doctor	136 (89.9)	129 (86.6)	1.99	0.57
		Parents	1 (0.7)	2 (1.3)		
		Children	3 (2.0)	1 (0.7)		
		Spouse	13 (8.5)	17 (11.7)		
14	I want the following people to give me the opportunity to ask questions.	Doctor	148 (96.7)	135 (90.6)	10.02	0.02
		Parents	1 (0.7)	2 (1.3)		
		Children	2 (1.3)	0 (0)		
		Spouse	2 (1.3)	12(8.1)		
15	I want the following people to inform me about possible therapies.	Doctor	146 (95.4)	139 (93.3)	3.38	0.38
		Parents	2 (1.3)	1 (0.7)		
		Children	1 (0.7)	0 (0)		
		Spouse	4 (2.6)	6 (6.0)		
16	I want the following people to inform me about alternative treatment methods (traditional therapy, palliative therapy).	Doctor	143 (93.5)	137 (91.9)	1.68	0.64
		Parents	3 (2.0)	3 (2.0)		
		Children	1 (0.7)	0 (0)		
		Spouse	6 (3.9)	9(6.0)		
17	I want the following people to inform me about the effects of cancer on life circumstances.	Doctor	136 (88.9)	131 (87.9)	7.71	0.05
		Parents	6 (3.9)	2 (1.3)		
		Children	3 (2.0)	0 (0)		
		Spouse	8 (5.2)	16 (10.3)		
18	I want the following people to characterize the expected course of disease in all clarity.	Doctor	145 (94.8)	137 (91.9)	6.08	0.11
		Parents	1 (0.7)	2 (1.3)		
		Children	3 (2.0)	0 (0)		
		Spouse	4 (2.6)	10 (6.7)		

### 3.4. Relation between general attitudes and the personal wish for knowledge

To investigate the association between attitudes regarding the knowledge of having cancer in general and if the person had cancer himself/herself, a Spearman-Brown correlation was applied, showing a small but significant correlation (*r* = 0.270, *p* < 0.001). The result indicates a positive and significant correlation between question 1 (It is better that the patient knows that he/she has cancer) and question 10 (I want to know that I have cancer) of the questionnaire. It seems that the more people agree that they should inform the cancer patient about their disease, the more inclined they are to want to be informed themselves if they were to have the disease.

To assess the moderating influence of gender, age, education, caregiver status, and the general attitude “It is better that the patient knows that he/she has cancer” on the statement “I want to know that I have cancer”, logistic regressions were conducted using the PROCESS procedure (Hayes, 2013). The results, detailed in Table 3, demonstrated a significant impact of education (*p* < 0.05) and personal history (*p* < 0.001) on the desire to be informed about cancer. However, no significant effects were observed for gender and age.

**Table 3:** Logistic regression of the possible moderators (age, gender, education and caregiver status) to the general attitude (It is better that the patient knows that he/she has cancer) on the individual wish to know about cancer (I want to know if I have cancer) (*n* = 302).

Moderator	Variables	model	*p*-value	B (SE)	*p*	CI 95%
Age	Age	21.16	0.00	−0.02 (0.02)	0.23	−0.07–0.01^(n.s)^
	General attitude			1.59 (0.35)	0.00***	0.89–2.29
	Interaction			−0.07 (0.04)	0.14	−0.15–0.02^(n.s)^
Sex	Sex	20.23	0.00	−0.15 (0.41)	0.71	−0.94–0.65^(n.s)^
	General attitude			1.5 (0.36)	0.00***	0.81–2.22
	Interaction			0.99 (0.91)	0.27	−0.79–2.77^(n.s)^
Education	Education	25.36	0.00	−0.31 (0.14)	0.03*	−0.59–0.03
	General attitude			1.64 (0.36)	0.00***	0.92–2.36
	Interaction			−0.49 (0.35)	0.16	−1.17–0.20^(n.s)^
Caregiver	Caregiver	70.02	0.00	−2.31 (0.43)	0.00***	−3.15–1.47
	General attitude			2.62 (0.61)	0.00***	1.46–3.82
	Interaction			2.13 (0.67)	0.00***	1.29–4.55

SE (standard error), *p < 0.05, **p < 0.01, ***p < 0.001, LLCI (lower level confidence interval), ULCI (upper level confidence interval). 95% CI that does not include zero.

In scrutinizing the moderating roles of gender, age, education, family history of cancer, and the general attitude toward delivering challenging news, the reported confidence intervals (CIs) indicated that only the interaction between caregiving status and the general attitude was statistically significant in predicting an individual’s preference for being informed about their cancer diagnosis (95% CI = [1.29–4.55]; the 95% CI did not encompass zero). Conversely, the interactions of gender, age, education, and the general attitude were not significant (the 95% CIs included zero). Thus, caregiving for a cancer patient plays a noteworthy moderating role in this relationship.

These results illustrate a distinction in the relationship between responding to the first item (“It is better that the patient knows that he/she has cancer”) and the tenths item (“I want to know that I have cancer”) in the two groups. Specifically, the association between the general attitude toward awareness of the illness and the personal wish to be informed was more robust in the caregiver group.

### 3.5. Talking about cancer with patients

From 153 caregivers, we received answers from 127 respondents about the following questions: “Does anybody talk with the patient about his/her cancer?” and “Who was the person to break the bad news?”. Table 4 shows the results; 46 (36.2%) patients were spoken to directly about their illness, but nobody had ever talked with 81 (63.8%) patients about their cancer.

**Table 4:** Caregivers’ conversations with the patients (n = 127).

The patient was NOT spoken about to his/her cancer	81 (63.8%)
The patient was spoken to about his/her cancer	46 (36.2%)
*The informant*	
Physician	35 (76.1%)
Spouse	4 (8.7)
Siblings	4 (8.7%)
Parents	0 (0%)
Friend	3 (6.5%)

## 4. Discussion and conclusion

The study aimed to assess the attitudes of both caregivers of cancer patients and non-caregivers toward communicating with individuals diagnosed with cancer. It examined the alignment between general attitudes toward delivering difficult news in cancer communication and participants’ personal preferences if they were themselves diagnosed with cancer. Moreover, the study investigated the extent to which caregivers engaged in conversations with patients, including who assumed the responsibility of conveying the diagnosis. In summary, the majority of participants affirmed the patient’s right to be informed about the diagnosis and prognosis, expressing a personal desire for the same transparency if they were facing a cancer diagnosis. However, a significant portion of caregivers reported not having discussed the illness with the affected patient.

The first finding of this study revealed that nearly all participants, regardless of their caregiving status, advocated for direct communication with patients and expressed a desire to be informed if they were diagnosed with cancer. Many persons express a strong desire to possess knowledge about their diagnosis (Jung et al., 2019). This sentiment aligns with the principles of biomedical ethics as outlined by Beauchamp and Childress (2001), emphasizing the “right to know” in numerous studies. However, half of the participants did not support immediate disclosure during the initial conversation. This is while studies have shown that it is better to share information with the patients as much as they understand and gradually over several sessions with empathy (Zendehdel, 2019).

One particularly intriguing finding is that 92% of caregivers expressed a preference for being informed if they were diagnosed with cancer, while only 65% of non-caregivers shared this sentiment. This observation mirrors the existing literature on information avoidance, wherein some individuals choose to remain uninformed, especially when they anticipate that the information may negatively impact their mental well-being. Case et al. (2005) posited that individuals may, at times, opt not to confront the reality of their situation, particularly in matters of health. They argued that information avoidance is frequently linked to feelings of anxiety, fear, self-efficacy, and locus of control. Consequently, while it is generally assumed that information can alleviate anxiety, this does not hold true for all individuals. Even in Western countries like the USA, a nationally representative sample demonstrated that 4 to 5 out of 10 patients actively avoided information. This behavior was attributed to factors such as gender (male), a family history of cancer, and feelings of information overload (Chae et al., 2020).

Furthermore, the findings show that healthcare professionals were the most preferred source for delivering the bad news among both groups. Only in item 14 did some people in the non-caregivers group state that they preferred their spouse to give them the opportunity to ask questions. Selecting the spouse as the source of questions and answers may show the need for people to communicate during a chronic illness; however, this answer was significantly higher among non-caregivers. People who had dealt with cancer in their family preferred professional sources for questions and answers. Nevertheless, an Iranian study found, that only 32 (13.6%) of the medical staff had received training in delivering bad news, and a significant majority, 195 (83%), expressed the need for a course to develop this skill (Biazar et al., 2019). This highlights a clear gap in training and underscores the importance of providing further education in this area especially because clinical communication encompasses far more than the mere transmission of information (Bousquet et al., 2015; Matthews et al., 2019; Tranberg and Brodin, 2023).

The study’s most surprising discovery was the contrast between the positive attitudes toward direct communication and the actual practices observed. A majority of caregivers reported that no one had discussed the cancer diagnosis with the patients. As a result, they were uncertain whether the patient was aware of their condition or not. This pattern aligns with previous Iranian studies, which consistently found that the majority of patients remained unaware of their diagnosis (Zahedi and Larijani, 2009; Parsa et al., 2011; Lashkarizadeh et al., 2012; Joibari et al., 2013). Similar trends have been observed in Eastern countries. For instance, a study in Lebanon revealed that although most participants believed patients should be informed about their disease, but only 14% of physicians disclosed the truth (Farhat et al., 2015). In China, Hahne et al. (2020) also noted that doctors typically inform the family first, and if requested, withhold information from the patient. In two other Chinese studies, between 35 and 50% of doctors said that they withhold information about cancer from their patients (Fan et al., 2011; Wang et al., 2011).

The fact that over 63% of our participants chose not to share information with their patients raises several critical concerns. Firstly, despite Iranian medical students being educated in biomedical ethics and the SPIKES protocol, in practice, many appear to defer to the families’ wishes to avoid direct communication with patients. Secondly, due to this lack of direct communication, there is no way to ascertain whether patients are aware of their cancer and what their specific needs might be. Consequently, patients may not be adequately included in the decision-making process regarding their treatment. Fourthly, it appears that families’ expectations serve as a barrier to open and comprehensive communication between healthcare professionals and cancer patients. In Iran, the role of the family is paramount in handling bad news and providing care (Bazrafshan et al., 2022). In many cases, it is the family that determines how much patients are told, who delivers the information, and even when to transition from treatment to palliative care (Shah et al., 2023). Abazari et al. (2017) created a localized protocol to break bad news in Iran. They emphasized including the views of close family in informing the patient, prioritizing the “no harm” principle over “respect for autonomy”, replacing the term cancer with less scary words, planning and preparing the family to tell the truth, and not mentioning a potential time of death. Part of this recommendation seems not to be in line with the western view on delivering bad news but may work better in Iran (Scheidt et al., 2017).

There are some limitations to this study. One of the limitations of the research was the lack of qualitative investigation of this issue. Therefore, the information about this issue was collected only quantitatively. The majority of respondent were female. Although it is not surprising as female consists of the majority of caregivers around the world (Sharma et al., 2016), the generalization of the study results to the male caregivers in Iran should be done with caution. The majority of caregivers in this study took care of an elderly patient and withholding the information from them was possible. Considering the level of access to information, similar study with young population with cancer would have different result in Iran. Moreover, non-caregivers were recruited via social media, whereas caregivers were recruited via social media and phone calls (NGO contact). Thus, a selection bias cannot be completely excluded. Nevertheless, the majority of the second group also answered the online version of the questionnaire, and there was no difference in education and almost no difference in age (caregivers: M = 45.1 years, SD = 6.3 versus non-caregivers: M = 42.8 years, SD = 6.1), which indicates the comparability of the two groups.

In conclusion, this study analyzed the general and individual attitudes of caregivers and non-caregivers regarding communication with cancer patients. It also looked at the congruency of the answers with a scenario of having cancer in the future and the wish to know about it. The study explored how many participants had actually talked with the cancer patients in their family. The majority of participants believed that patients have the right to know about the diagnosis and prognosis, and they wished to know it if they ever had cancer. However, the majority of caregivers stated that they have not talked with the cancer patient about their illness.

These findings may have some implications for families, patients, healthcare services, and policymakers. For families and the patients, it is important to know that the “right to know” should not be withheld based only on the expectation that the patient will lose hope and the spirit to fight cancer. Instead, giving the right to “not know” can be achieved by asking the patient how much they want to know. Healthcare professionals may face conflicts between the protocols of breaking bad news and the wish of the family to not directly talk with patients. Healthcare staff need to consider the cultural background of the patient and find a way to “not harm” the therapeutic relationship with the family while informing the patient of the disease. Policymakers should create a curriculum to deliver bad news in a culturally competent way and to facilitate a patient’s need to express their emotions and needs.

## Data availability statement

The raw data supporting the conclusions of this article will be made available by the authors, without undue reservation.

## Ethics statement

The studies involving humans were approved by the University of Isfahan Ethics Committee. The studies were conducted in accordance with the local legislation and institutional requirements. The participants provided their written informed consent to participate in this study.

## Author contributions

AN and SS were involved in data collection. AN, SS, WR, and PB contributed in analyses and writing up the report. All authors contributed to the article and approved the submitted version.
